# Cultural change and loss of ethnoecological knowledge among the Isthmus Zapotecs of Mexico

**DOI:** 10.1186/1746-4269-9-40

**Published:** 2013-06-11

**Authors:** Alfredo Saynes-Vásquez, Javier Caballero, Jorge A Meave, Fernando Chiang

**Affiliations:** 1 Jardín Botánico, Instituto de Biología, Universidad Nacional Autónoma de México, Apdo, Postal 70-614, CP 04510 Ciudad Universitaria, DF, México; 2 Departamento de Ecología y Recursos Naturales, Facultad de Ciencias, Universidad Nacional Autónoma de México, México 04510DF, México; 3 Departamento de Botánica, Instituto de Biología, Universidad Nacional Autónoma de México, México 04510DF, México

**Keywords:** Cultural change, Traditional ecological knowledge, Zapotecs, Tehuantepec Isthmus

## Abstract

**Background:**

Global changes that affect local societies may cause the loss of ecological knowledge. The process of cultural change in Zapotec communities of the Oaxacan Isthmus intensified during the first years of the 20^th^ century due to industrial and agro-industrial modernization projects and an increase in the level of formal schooling. Based on the case of the Oaxacan Isthmus, this study assesses the relationship between cultural change and the loss of traditional ecological knowledge (TEK).

**Methods:**

Three hundred male heads of family were interviewed from three municipalities in the Isthmus of Tehuantepec, Oaxaca, Mexico selected to span a wide range of cultural change. Each participant was shown herbarium specimens and photographs of a sample of 30 species drawn from a pool of 94 representing local plant diversity. Visual recognition of each species, knowledge of plant form, generic name, specific name, and local uses were scored. The sum of the five scores provided an index of global knowledge which we used as a proxy for TEK. Analysis of variance revealed differences between groups of economic activities. We collected socio-demographic data from the interviewees such as age, level of schooling, and competency in the local language. With these data we ran a principal component analysis and took the first axis as an index of cultural change, and correlated it with the scores obtained each respondent.

**Results:**

We found statistically significant differences between groups of people with different economic activities, as well as a highly significant negative relationship between the Index of cultural change and ecological knowledge at all levels, with regression coefficients between 81.2% and 88.3%, indicating that cultural change is affecting traditional botanical knowledge.

**Conclusions:**

Our results shown that cultural change, as indicated by occupational activity, level of formal schooling, and competence in the indigenous language, is negatively associated with the loss of Zapotec ethnobotanical knowledge. Heads of family engaged in secondary economic activities and services were less culturally competent, especially regarding the knowledge of generic and specific names as well as plant uses.

## Background

Knowledge of plants and animals is a reflection of the relationship between human communities and the physical, biotic, and cultural environment through time [1,2]. Economic development and current systems of formal education have transformed these relations. As a result, even though these systems of education and economic development have contributed to an increase in the material well-being of these societies, they have also led to loss of traditional ecological knowledge [3-7].

Cultural change is a multifaceted process that may often includes the acquisition of urban tastes and values by rural societies, the loss of local languages, and the industrialization or abandonment of primary production or agricultural practices. This process generally leads to a corresponding loss of traditional ecological knowledge [8]. Several studies show that monolingualism, occupation and the degree of schooling are the most important indicators of cultural change [5,8-15].

Traditional Ecological Knowledge (TEK) refers to the knowledge, beliefs, and practices that have to do with the relationship that human societies have with their natural surroundings [16]. The development of TEK is a dynamic process, and responds to natural and historical conditions of each society [17]. Therefore, if natural and social conditions in a community change, TEK should also be affected. An example of a social change is the loss of indigenous languages. The rate and severity of this change varies between communities, and some have shown a surprising resistance to the loss of their native tongue, as has been seen with the Zapotec people from the Isthmus of Tehuantepec in the state of Oaxaca, Mexico.

Among the most conspicuous expressions of TEK are the biological classifications and nomenclatures developed by local cultures, in which groups of living organisms are named and organized within a specific cultural environment. These groups of organisms are called *taxa* and some of these categories have a close correspondence with scientific biological classification [18-24]. Berlin [25] suggests that the ethnobiological vocabulary is eroded when activities most related to the environment lose importance.

The process of erosion of traditional classifications also seems to be negatively associated to a higher degree of formal education of native speakers. In Venezuela, Zent [13] documented the negative relationship between the increase of schooling and the bilingual skills of villagers in the local Piaora language and Spanish, which resulted in the loss of knowledge of plant names at the generic level. In rural Oaxaca, Mexico, Cortés-González [26] found that formal education was also a variable strongly negatively associated with the loss of local botanical knowledge. However, little is known about how other factors such as a change in occupation and age join formal education and competencies in native languages to explain the loss of TEK. The present study situated at the Isthmus of Tehuantepec, Oaxaca, Mexico, analyzed the relationship between that cultural change resulting from change in occupation, increase in formal education in Spanish, and the predominance of the Spanish language and decrease in knowledge of names and uses of plants. We chose the region of the Isthmus of Tehuantepec as a study area because it presents a locale with both a strong persistence of local culture and language, as well as containing different degrees of cultural change. We expected that that the populations with greater cultural change would have less ethnobotanical skills than the more culturally conservative populations. The impact of cultural change on the loss of traditional botanical knowledge was assessed through a statistical analysis of correlation between botanical knowledge and an index of cultural change that includes various socio-demographic variables.

## Methods

### Study area

**Figure 1:**
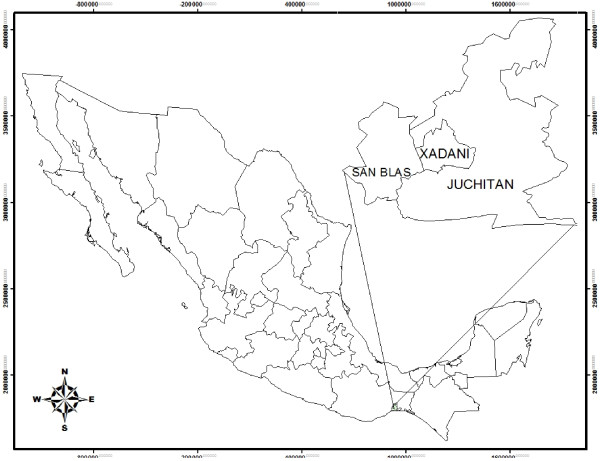
Location of study sites in Oaxaca, Mexico.

Data were collected in three Zapotec communities in the municipalities of Juchitán de Zaragoza (16° 25’ 58.4” N, 95° 01’ 19.1” W), San Blas Atempa (16° 19’ 36.6” N, 95° 13’ 38.9” W), and Santa María Xadani (16° 21’ 36.3” N, 95° 01’11.18” W), in the state of Oaxaca (Figure 1).

In this area, the local language is Isthmus Zapotec (which belongs to the great Otomanguean family that includes Mixtec, Amuzgo, and Chatino, among others) [27,28]. More than 70% of the population speaks the indigenous language [29] (Table 1), with slight dialect variations between municipalities.

**Table 1:** Sociodemographic data of the three municipalities

**Municipality**	**% Speaking indigenous language**	**% Speaking Zapotec monolingual**	**% Speaking Zapotec monolingual**	**% Of 15 year-old or older who read and write in Spanish**	**Average number of years in school**	**% Of the population engaged in primary production activities**
	**Year 2000**	**Year 2000**	**Year 1950**	**Year 2000**	**Year 2000**	**Year 2000**
Juchitán de Zaragoza	70.01	8.81	27.26	52.11	6.26	13.89
San Blas Atempa	92.76	24.87	72.08	39.95	3.56	33.79
Santa María Xadani	97.71	34.07	83.33	32.05	3.36	35.01

Linguistic competencies, level of schooling and primary production as an occupation of the three municipalities. The gradient is shown in percentages of Zapotec and monolingual speakers, reading and writing skills in Spanish, formal education level and population engaged in primary productivity activities.

The proportion of the population that speaks an indigenous language in these Zapotec communities is relatively high compared to the average in other indigenous regions in Mexico. Nevertheless, in 1950 the percentage of people speaking Zapotec only (monolingual) was much higher than in 2000. In Juchitán, 27.3% spoke the language in 1950 as compared to 8.8% in 2000. In San Blas Atempa, these percentages were 72.1% and 24.9% respectively, and in Santa María Xadani 83.3% and 34.1%. These numbers illustrate the speed with which linguistic patterns are changing [29-31]. These towns should have different levels of cultural change, and therefore provided the variation in cultural impact that our study required.

### Fieldwork and data analysis

One hundred male family heads, divided in two groups of 50 individuals based on occupation, were randomly selected in each municipality. The first group was made up of people involved mainly in primary economic activities, which involve natural resource extraction and management, including subsistence farmers, hunters, and loggers. The second was made up of people mainly involved in secondary and tertiary level economic activities. Secondary level activities are those involving processing primary activity products, whereas tertiary activities are the providing of services. This group included construction workers, dealers/traders as well as professionals with a college education such as physicians, engineers, and schoolteachers. This study did not involved any kind of experimental research with human subjects. Interviewing local peoples was authorized by the municipal authorities in each locality. Each interview was carried with previous informed consent of the interviewees.

Based on previous studies that suggest [7,13] that most traditional knowledge is acquired before the age of 30, the average age of the interviewees selected for this study was 48.6 years old. We assumed that all interviewees may have had the same level of knowledge. All interviewees who participated in this study gave their previous informed consent to participate. Each of the 300 semi-structured interviews was conducted in Zapotec by the first author, a native Zapotec speaker. When the interviewees were not competent in Zapotec the interview was conducted in Spanish. We obtained the respondent’s personal information including age, main occupational activity, number of years of formal education, and language proficiency. For language competency, we used a modified Zent [12] scale consisting of values 1 and 0 (yes/no) as an answer to the following three questions: (1) Do you speak Zapotec? (2) Do you understand Zapotec? (3) Do you speak and understand Spanish?

To assess their knowledge of botanical nomenclature and plant uses, each family head was shown stimuli consisting of both herbarium material and a good quality picture of a set of flowers, stems, and fruits of different local plants. The plants used as stimuli were selected from 30 sampling sites (50 m by 2 m = 100 m^2^) within a well-preserved forested area shared by the three communities. A sample of 30 out of the 94 plant species recorded in the sampling sites were selected in groups of six, representing the five life forms recognized: trees (yaga), shrubs (yaga hunni), vines (luba'), herbs (guixi), and a category locally known as “guie” (“flower” in Zapotec). Each group of six included species with high, medium, and low values of ecological importance. The exception was the herb category. Herbs were randomly chosen from a list of plant names collected in the area (Table 2). The purpose of this selection procedure was to reflect the diversity of growth forms and the different values of relative ecological importance represented, as well as to reduce personal biases in choosing the plants. The Zapotec names of the plants species included in the sample were already known from previous ethnobotanical fieldwork conducted by the first author in the three municipalities.

**Table 2:** Species selected for the study

**Family**	**Species**	**Zapotec name**
Amaranthaceae	*Amaranthus spinosus* L.	Balaadxi gui'chi' (gr)
Apocynaceae	*Marsdenia coulteri* Hemsl.	Luba' biñaa (v)
Apocynaceae	*Plumeria rubra* L.	Guie' chaachi (gu)
Bignoniaceae	*Crescentia alata* Kunth	Bitu xhiga gui'xhi' (s)
Bignoniaceae	*Mansoa hymenaea* (DC.) A.H. Gentry	Luba' bete (v)
Burseraceae	*Bursera schlechtendalii* Engel.	Yalaguitu (t)
Combretaceae	*Combretum fruticosum* (Loefl.) Stuntz	Luba' begu (v)
Combretaceae	*Polanisia viscosa* L. (DC.)	Stoope gui'xhi' (gr)
Commelinaceae	*Commelina coelestis* Willd.	Guie'duza (gu)
Cucurbitaceae	*Ibervillea* sp.	Luba' cuba, Luba' manzanina, Luba' melón gui'xhi' (v)
Euphorbiaceae	*Croton niveus* Jacq.	Copachil (s)
Euphorbiaceae	*Euphorbia* sp.	Pichinchi yuu (gr)
Hippocrateaceae	*Hippocratea excelsa* Kunth	Luba' biichi (v)
Julianaceae	*Amphipterygium adstringens* (Schltdl.) Standl.	Yaga yala (t)
Leguminosae	*Aeschynomene americana* L.	Yaga tama (s)
Leguminosae	*Apoplanesia paniculata* C. Presl.	Guie' bi'chi' (gu)
Leguminosae	*Diphysa minutifolia* Rose	Guiiña' bidxi (s)
Leguminosae	*Lonchocarpus sericeus* (Poir.) Kunth ex DC.	Guie' gade (gu)
Leguminosae	*Microlobius foetidus* (Jacq.) M. Sousa & G. Andrade	Biquiiche dxa' (t)
Leguminosae	*Mimosa acantholoba* (Humb. & Bonpl. ex Willd.) Poir.	Chumaaga o Guichi xhi gueza (s)
Leguminosae	*Senna atomaria* (L.) H. S. Irwin & Barneby	Beza duni (t)
Leguminosae	*Senna skinneri* (Benth.) H.S. Irwin & Barneby	Guie' bizu, Bara seda (gu)
Leguminosae	*Prosopis juliflora* (Sw.) DC.	Yaga bii (t)
Malpighiaceae	*Malpighia emarginata* Sessé et Moc. ex DC.	Combriu (s)
Malvaceae	*Hibiscus* sp.	Xiaa gui'xhi' (gr)
Portulacaceae	*Portulaca oleracea* L.	Xedxe (gr)
Rhamnaceae	*Ziziphus amole* (Sessé & Moc.) M. C. Johnst.	Xuba beza (t)
Sapindaceae	*Serjania goniocarpa* Radlk.	Luba' golondrina (v)
Scrophulariaceae	*Capraria biflora* L.	Bitiaa gui' xhi' (gr)
Theophrastaceae	*Jacquinia pungens* Gray	Guie' zee (gu)

*t* tree, *s* shrub, *v* vine, *gr* grass, *gu* “guie'”.

We asked each respondent five questions: (1) Do you recognize the plant? (2) Do you recognize the plant form? (3) Do you know the generic epithet? (4) Do you know the specific epithet? And (5) do you know if the plants are used, and if so, which parts are used and for what purposes? We also tried to assess whether respondents had any additional knowledge regarding the plants’ biology, habitat, flowering season, etc.

The field method based on ecological sampling for selecting the stimuli, the high number of interviewees, the use of the native language during interviewing, as well as the previous knowledge of the Zapotec names for plants, were all factors aimed at reducing the risk of bias in the answers to the questionnaire. With regard to the classification of people by economic activity, we are aware of the risk of misclassification due to the changes in activity that may occur over time. However, we found that the discriminant analysis on sociodemographic data (not shown here) supports the grouping used in this research.

For data analysis all positive answers were summed, expressed as ratios and transformed according to Freeman and Tukey [32] to normalize them. An analysis of variance (ANOVA) was carried out to find statistical differences between groups of people according to their economic activity. By adding the scores for each of the above five levels of knowledge from each respondent, we calculated a global index of ethnobotanical knowledge or competence for that respondent using the following formula: Global Index = ∑ CVR + CPF + CRG + CRS + CRU, where CVR = competence in visual recognition; CPF = competence in recognition of plant form; CRG = competence in recognition of genera; CRS = competence in recognition of specific name, and C RU = competence in knowledge of use.

We used a principal component analysis (PCA) to calculate the index of cultural change using the respondent’s personal information as variables (age, main occupational activity, years of formal schooling, municipality, and language proficiency in Spanish and Zapotec). We used the score of each person interviewed along the first principal component directly as index values of cultural change (Table 3).

**Table 3:** Results of the Principal Component Analysis (PCA) of the 300 heads of family

**Variable**	**Component 1**
Municipality	0.106
Occupation	**0.780**
Age	−0.343
Schooling	**0.812**
Understands Spanish	**0.712**
Speaks Spanish	**0.831**
Speaks Zapotec	−0.108

The loadings of the most important social and demographic variables are indicated in bold characters. Extraction Method: Principal Component Analysis. Rotation Method: Varimax Normalization with Kaiser. Rotation converged in 3 iterations.

To correlate cultural change and ethnobotanical knowledge, a linear regression analysis was carried out using the ethnobotanical competence scores of the interviewees and the index of cultural change. SPSS v. 15.0 and Statistica v. 7 were used for all analyses.

## Results

### Social and demographic variables

**Figure 2:**
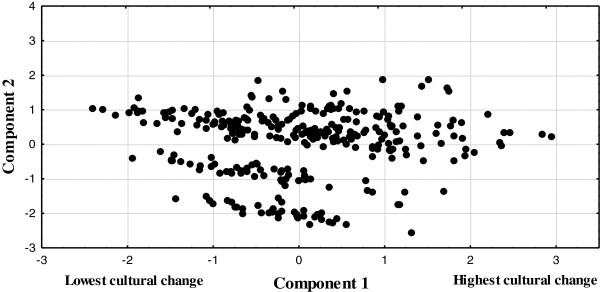
**Ordination of the 300 interviewed heads of family, according to seven social and demographic variables.** Subsistence workers with a low degree of formal education and less fluency in Spanish are located on the extreme left side of the classification axis (negative values), while the interviewees involved in secondary and tertiary activities were located on the opposite extreme (positive values).

When the 300 interviewees were compared by the social and demographic variables using, we obtained a cultural change gradient along the first principal component that explained 38% of total variation. The highest loadings were for occupational activity, fluency in Spanish (when Spanish is spoken and understood), and level of schooling; for the second component, locality and whether Zapotec was spoken or not were the most important factors (Table 3). Subsistence workers with a low degree of formal education and less fluency in Spanish were located on the extreme left side of the classification axis (negative values), while the interviewees involved in secondary and tertiary activities were located at the opposite extreme (positive values). The first group thus represents the most culturally conservative subjects, whereas the last ones were those with the highest degree of cultural change (Figure 2). Each interviewee’s coordinate along the first axis of the principal component was interpreted as the index of cultural change.

### Knowledge of Zapotec botanical nomenclature and local uses

Table 4 shows the descriptive statistics of the five levels of botanical knowledge on a regional scale (300 interviewees). A relatively high coefficient of variation can be observed, indicating high data dispersion. Variability is higher as knowledge of nomenclature becomes more specific. Nevertheless, when the global index is evaluated, variability decreased although it remains relatively high (33.57%). It was also observed that the competence on the local botanical knowledge is lower in the group of secondary and tertiary activity in all the levels analyzed, especially at the generic, specific names, and knowledge of uses (Table 4).

**Table 4:** Statistics of the levels of knowledge at regional scale

		**Competence in visual recognition**	**Competence in recognition of plant form**	**Competence in recognition of genera**	**Competence in recognition of specific name**	**Competence in knowledge of use**	**Global index**
**Primary activity**	N	150	150	150	150	150	150
	Mean	**28.22**	**28.13**	**26.48**	**25.29**	**23.80**	**131.9200**
	Std. Desv.	1.706	1.773	2.548	3.428	2.761	10.91119
**Secondary and tertiary activity**	N	150	150	150	150	150	150
	Mean	**19.23**	**18.65**	**14.71**	**11.75**	**12.65**	**76.9867**
	Std. Desv.	5.476	5.796	6.471	6.646	5.682	28.80669
**Total**	N	300	300	300	300	300	300
	Mean	**23.72**	**23.39**	**20.59**	**18.52**	**18.22**	**104.4533**
	Std. Desv.	6.057	6.389	7.673	8.594	7.148	35.06842
	Coefficient of variation (%)	**25.53**	**27.31**	**37.26**	**46.40**	**39.23**	**33.57**

Means, standard deviations and coefficients of variation are shown for each level of knowledge. *N* number of interviewees;

*Std. dev.* standard deviation.

There was a statistically significant difference between the two groups of occupational activities, in all levels of knowledge of local botanical nomenclature, and the knowledge of uses as determined by one-way ANOVA (*F* = 456.531, *P* = *0.000*; *F* = 457.22, P = 0.000; *F* = 431.136, P = 0.000; *F* = 432.363, P = 0.000; *F* = 458.053, P = 0.000 and *F* = 565.941, P = 0.000) (Table 5).

**Table 5:** Comparison between groups of activities in all levels of knowledge

**Level of knowledge**		**Sum of squares**	**df**	**Mean square**	**F**	**Sig.**
**Competence in visual recognition**	Between groups	12631.781	1	12631.781	456.531	**<0.001**
	Within groups	8245.385	298	27.669		
	Total	20877.166	299			
**Competence in recognition of plant form**	Between groups	13457.411	1	13457.411	457.220	**<0.001**
	Within groups	8771.075	298	29.433		
	Total	22228.486	299			
**Competence in recognition of genera**	Between groups	14436.678	1	14436.678	431.136	**<0.001**
	Within groups	9978.589	298	33.485		
	Total	24415.267	299			
**Competence in recognition of specific name**	Between groups	17385.702	1	17385.702	432.363	**<0.001**
	Within groups	11982.854	298	40.211		
	Total	29368.556	299			
**Competence in knowledge of use**	Between groups	9706.413	1	9706.413	458.053	**<0.001**
	Within groups	6314.800	298	21.191		
	Total	16021.213	299			
**Global index**	Between groups	4905.139	1	4905.139	565.941	**<0.001**
	Within groups	2582.835	298	8.667		
	Total	7487.974	299			

The analyses of variance show a statistically significant difference (indicated in bold) in all levels of knowledge.

**Figure 3:**
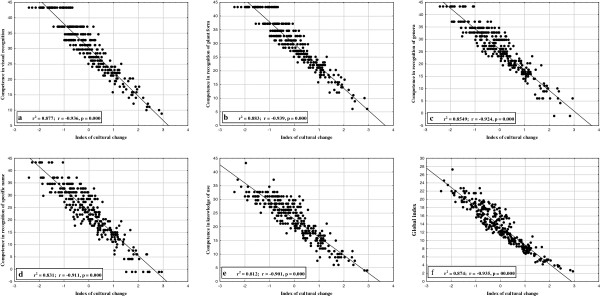
**Regressions on the Index of cultural change of all the levels of competences of knowledges. a)** Competence in visual recognition, **b)** Competence in recognition of plant form, **c)** Competence in recognition of generic name, **d)** Competence in recognition of specific name, **e)** Competence in knowledge of uses and **f)** Global index (integrating the last five).

Knowledge of Zapotec botanical nomenclature from visual recognition was influenced by cultural change. We observed that subsistence workers on the extreme left side of the graph (Figure 2) had the highest scores of knowledge, while those showing the highest cultural change, on the right of the graph, had the lowest scores (*F* = 1175.274, *P* = 0.000, *R*^2^ = 87.75%; Figure 3a). Only a few of the interviewees, agricultural subsistence farmers, had similar scores to that of the workers engaged in secondary or tertiary activities. Something similar happened between some workers engaged in secondary and tertiary activities, whose scores were similar to that of peasants

The same pattern of decreasing the ethnobotanical knowledge as cultural change increases was also seen in knowledge of plant form (Figure 3b) in which we saw a decrease in ability to identify the plant type as the index of cultural change increased (*F* = 2252.49, *P* = 0.000, *R*^2^ = 88.30%).

With regard to knowledge of genera, the behavior of the interviewees is very similar to the previous categories of knowledge. This implies a decrease in their ethnobotanical competence as cultural change increases (*F* = 1756.226, *P* = 0.000, *R*^2^ = 85.49%; Figure 3c). When knowledge at the species level and the cultural change index were correlated, we found a similar pattern (*F* = 1472.019, *P* = 0.000, and *R*^2^ = 83.16%; Figure 3d). Knowledge of the uses of the 30 species also showed a decreasing pattern as the index of cultural change increased (*F* = 1289. 84, *P* = 0.000, *R*^2^ = 81.23%; Figure 3e).

By integrating the five levels of ethnobotanical knowledge (competence in visual recognition, competence in recognition of plant forms, competence in recognition of generic name, competence in recognition of specific name, and competence in knowledge of uses), we calculated a global knowledge index which also showed a tendency to decrease when cultural change increased, as can be seen in Figure 3f (*F* = 2073.40, *P* = 0.000, *R*^2^ = 87.43%).

## Discussion

Our results show a strong negative relationship between cultural change and ethnobotanical competence in all of the evaluated aspects. The global knowledge index that encompasses the five analyzed levels of botanical knowledge showed a strong negative correlation with the rate of cultural change. This finding coincides with Zent´s [12,13] results from Venezuela. Therefore, cultural change experienced by the Zapotec people from the Isthmus of Tehuantepec as a result of regional development processes is related with the deterioration of Traditional Ecological Knowledge.

The agricultural and industrial modernization processes promoted by the state were production oriented, and they polarized—economically and politically— society and modified the cultural reproduction conditions of the traditional [33]. One of the most obvious impacts of this modernization is that a significant proportion of the population shifted from primary activities to secondary economic activities and services. Between 1950 and 1960, 70-75% of the economically active population was engaged in subsistence farming [34]. By 2000, only 35% or less was still engaged in primary activities [29]. This might have been the result of the construction of the Benito Juarez dam in the late fifties, when agriculture underwent a process of modernization accompanied by tenure conflicts and widespread sale of land [35].

As a result of the change in occupational activities, a large group of the population has had less contact with natural surroundings. We hypothesize that these new social circumstances are related to the decrease in local botanical knowledge. Supporting this notion is our observation that respondents engaged in secondary activities and services were less competent at all levels of the ethnobotanical knowledge, especially regarding generic and specific names of plants.

Despite this general decline in local knowledge about plants, there are species in the study area that have never been named in Spanish. This is the case of some species that still have a social function, and are part of everyday life for most people and, as a consequence, everyone, even those from outside the community, recognizes these species by their Zapotec name. On the other hand exposure to the natural environment associated with the sport hunting or countryside activities generates a certain visual knowledge, but not of the generic and/or specific names of the plants, as the time spent hunting is relatively brief and sporadic and hunters are more interested in wildlife than in the local flora. This could also be explained in terms of increasing plant blindness, which is the inability to realize the importance of plants for the ecosystem and human life as described by Yorek *et al.*[36].

The results of this study agree with those of Zent [12,13] and Cortés-González [26] in that they underscore that age is also an important factor in the degree of knowledge people have about the plants of their environment. As was suggested by Garro [37] age is naturally associated with the process of knowledge acquisition, so even if exposed to cultural change, individuals growing up and aging in any social group have access to their local knowledge.

Finally, the increase in formal education and the disuse of the local language are strongly associated with a lower degree of local botanical knowledge as was reported by Martínez-Ballesté *et al.*[15] and Thompson [9] in the Peninsula of Yucatan. Apparently, the time a child spends in school is a time that he could have, potentially, spent on activities in the field. Time spent in school thus seems likely to reduce the opportunity for learning about the local flora. Moreover, the exclusive use of Spanish in school promotes the loss of the local indigenous language and affects the transmission of knowledge about plants.

Formal education programs also marginalize local knowledge and bring about a change in cultural attitudes that encourage a more urban lifestyle and the estrangement from nature with the consequent disinterest of the local natural environment and the loss of knowledge related to it. This process increased during the decade of 1930 with the founding of industrially oriented schools and teachers who were in charge of organizing the peasants to attend school and promote literacy campaigns [38].

The botanical knowledge that requires less cultural ability is visual recognition, as well as identification of plant form. Skills in visual recognition had the highest average among the respondents who also showed a greater consensus on this level as the coefficient of variation in this aspect was the smallest. Knowledge regarding visual appearance and plant form is acquired when the surrounding vegetation and/or gardens and home gardens are seen, even if the local language is not well known. However, the practice of walking and observing the plants in the countryside is also being lost, and local species from gardens, kitchen gardens, and orchards are being replaced by introduced species or removed for the construction of houses. Moreover, technological modernization together with incentives and facilities from the government increased the expansion of the agricultural frontier bringing about a decrease of native or original vegetation [34,35]. In this regard, it should be noted that the original area with original vegetation in the Isthmus of Oaxaca has been decreased. In 1970, there was 49.74% of native vegetation, whereas in 2000 the percentage was 35.17 [39]. Although culture is essentially conservative, it changes with time. However the modifications are slow and complex [40]. Our data show that cultural change (as indicated by occupational activity, level of formal schooling, competency in the local and national language, and locality) is related to the loss of Zapotec ethnobotanical knowledge. It is possible that other factors affecting cultural change and the loss of local botanical knowledge were not evaluated in this research. However, our study shows that it is possible to approach the dynamics of cultural change and the loss of ecological knowledge with a quantitative perspective that enables us to distinguish the characteristics of the phenomenon of loss and, therefore, the possible reacquisition of lost knowledge. Loss of TEK and the displacement of the local culture also involve the loss of founding myths, ontologies about the world, and ultimately a specific epistemology developed over hundreds of years [41].

## Conclusion

Our results show a strong negative relationship between cultural change and ethnobotanical competence in all of the evaluated aspects. The global knowledge index that encompasses general knowledge in the five analyzed levels of botanical knowledge shows a strong negative correlation with the rate of cultural change. Respondents engaged in secondary activities and services were less competent in all levels of the ethnobotanical knowledge tested, especially regarding generic and specific names of plants, and the knowledge of their uses.

Cultural change experienced by the Zapotec people from the Isthmus as a result of regional development processes is related with the deterioration in TEK. As a result of the change in occupational activities, a large group of the population have had less contact with the natural surroundings, which appears to have led to a significant decrease in local botanical knowledge.

Our data suggest that formal education programs marginalize local knowledge and bring about a change in cultural attitudes that encourage a more urban lifestyle, and the estrangement from nature with the consequent disinterest of the local natural environment. Schooling also contributes to this process by promoting the abandonment of the vernacular language, which is the fundamental instrument of transmission of local knowledge. Another important factor is that the object of ethnobotanical knowledge itself is rapidly being lost by the increasing deforestation that occurs in the area.

The change process described here should have not only an academic interest, but should also generate political and ethical reflections about the value of this knowledge for providing a basis to preserve and restore the local biodiversity.

## Abbreviations

TEK: Traditional ecological knowledge; PCA: Principal components analysis; ANOVA: Analysis of variance; Std. dev.: Standard deviation.

## Competing interests

The authors declare that they have no competing interests.

## Authors’ contributions

AS Conducted this study as a partial requirement to obtain the Ph.D. Degree in Biological Sciences at the Universidad Nacional Autónoma de México. He carried out the field survey, data analysis and interpretation and drafting the manuscript. JC along with AS conceived the study. He supervised its development and participated in the interpretation of results and drafting the manuscript. JM and FCh gave valuable insights for data gathering and analysis as well as for interpretation of results. All authors read and approved the final manuscript.

## Authors’ information

AS is currently a PhD (Biology) candidate at the Universidad Nacional Autónoma de México (UNAM).

JC: Biologist at the UNAM, Mexico, PhD at the University of California, Berkeley. Director the Botanical Garden UNAM, advising and conducting research on quantitative ethnobotany, non-timber forest products, traditional agroforestry systems, biological variation and evolution under domestication of useful plants.

JM: Full time Professor, Facultad de Ciencias, UNAM Research field: Biological sciences-plant sciences, vegetation structure, diversity and dynamics, vegetation functioning, biological conservation.

FCh: Researcher at the National Herbarium at the UNAM. Research field: Taxonomy of vascular plants (Mesoamerican Rutaceae), floristics of the Selva Lacandona and the region of Calakmul, Campeche, Mexico.
